# What’s the impact of voice-hearing experiences on the social relating of young people: A comparison between help-seeking young people who did and did not hear voices

**DOI:** 10.1371/journal.pone.0290641

**Published:** 2023-09-26

**Authors:** Aikaterini Rammou, Clio Berry, David Fowler, Mark Hayward

**Affiliations:** 1 School of Psychology, University of Sussex, Brighton, United Kingdom; 2 Research and Development Department, Sussex Partnership NHS Foundation Trust, Worthing, United Kingdom; 3 Brighton and Sussex Medical School, University of Sussex, Brighton, United Kingdom; University of Catania Libraries and Documentation Centre: Universita degli Studi di Catania, ITALY

## Abstract

Limited research has explored the specific impact of voice-hearing experiences upon the social relating of adolescents. This study examined the associations of voice-hearing in youth with social relating, and putative explanatory factors. An observational, cross-sectional design using a clinical comparison group was employed to examine historical and concurrent associations with voice-hearing. Thirty-four young people (age 14–18 years) with voice-hearing experiences and 34 young people who did not hear voices were recruited from NHS mental health services. Participants completed measures about social relating and potential explanatory factors. Analyses of covariance were used to examine between-group differences. Voice-hearers scored higher on negative schematic beliefs (self-beliefs, partial η^2^ = .163, p = .001; other-beliefs, partial η^2^ = .152, p =. 002) and depressive and anxiety symptoms (partial η^2^ = .23 and partial η^2^ = .24, p-s <. 001 respectively). The two groups did not differ significantly on childhood trauma levels (partial η^2^ = .02, p = .273), however, the voice-hearing group scored lower on premorbid adjustment (partial η^2^ = .19, p < .001). Hearing voices in help-seeking youth could be an indicator for social relating issues and holding negative schematic beliefs, and may be an indicator for of increased psychopathological complexity. Although poorer premorbid adjustment might indicate an early vulnerability to social relating difficulties, voice-hearing might be an aggravating factor and one that requires treatment.

## Introduction

Adolescence is an important time for the development and growth of social relationships [1], during which young people become more sensitive to others’ actual and perceived appraisals of themselves [2]. Research on psychotic experiences (PE), including hearing voices, shows that young people experiencing these phenomena may face difficulties to develop and maintain social relationships. Studies with young people at clinical risk for psychosis report lower levels of social support, greater social isolation [3, 4], less positive and more negative relationships with family members and friends, and higher levels of loneliness [5]; consistent with recent population research [6]. Young people who hear voices have also described being reluctant to disclose their voice-hearing experience due to stigma concerns, such as receiving unpleasant responses from others, which in turn could lead to social withdrawal [7]. This is problematic because social support may act in a protective capacity against PE and stress in adolescents [8]. Nevertheless, research focusing on voice-hearing in young people and its potential link with social relating aspects has been limited, with only a few recent studies looking into voice-hearing in clinical samples of young people [9–11].

### Voice-hearing as an indicator of and contributor to social disconnection

A small body of qualitative research with adult voice-hearers has indicated that voices could, for some individuals, serve a function, especially for those with depleted social networks and social contact, suggesting that voices might fulfil subjective social needs when these are not met by other social relationships [12]. Similar to adults, Parry, Loren and Varese [7] reported that young people commonly attributed their voice-hearing to loneliness and social isolation, highlighting the potential relational function of voices in youth. When voices were perceived as pleasant, young people described them as friends they could talk to, possibly addressing unmet social needs [13]. Young people, especially when experiencing low social belonging and connectedness [10], can become dependent upon interactions with voices [13], and thus voice-hearing could enhance feelings of isolation which can then exacerbate distress [7]. Similar findings have been found in adult literature with voice-hearing being considered socially disruptive, either directly, e.g., by interrupting conversations, saying things that undermine the trust in social others [14, 15], or indirectly by eliciting negative emotions, e.g., fear [15].

### Potential explanatory factors

The link between social relating difficulties and voice-hearing may be a dynamic, reciprocal process operating in both directions [16], and may be explained by adversity and psychopathology, General population studies have found that social functioning difficulties might be present before [17] and deteriorate during, and after, the onset of adolescent PE [18, 19], especially when PE become persistent [20]. Exposure to greater levels of trauma could predispose young people to form negative interpersonal schemas that could guide interpretation and response to interpersonal interactions [21] and in turn influence the social relationships of adolescent voice-hearers [22]. The association between childhood trauma and negative interpersonal schemas has been consistently found in adult literature [23], although evidence on the mediating role of schemas between trauma and voice-hearing is somewhat mixed [24, 25]. Nonetheless, negative schemas have been linked with hallucinations in community-based young adult research [26] and with the presence and severity of distressing PE in Child and Adolescent Mental Health Services (CAMHS) patients [27]. Thus, negative self-evaluations and negative comparisons of oneself to social others could have an adverse impact on young people’s relationships [28] and lead to disconnection from the social domain [29, 30].

Social difficulties could otherwise derive from greater symptomatology and its associations with functional impairment. Across four adolescent general population samples in Ireland [31], and a help-seeking clinical sample of young people [32], a dose–response relationship between risk for PE and the number of diagnosable psychiatric disorders was reported. However, clinical studies comparing young people with and without PE and with non-psychotic disorders indicate that those with PE presented with poorer global socio-occupational functioning even when the effect of multimorbidity [32, 33] and cognitive functioning, anxiety, depression levels and severity of psychiatric disorder was taken into account [34]. Therefore, social relating difficulties may be partially, but perhaps not fully, explained by voice-hearers tending to experience greater psychopathology in general.

### Aims

To explore the potential unique relationship between voice-hearing and social relating, we compared a clinical sample of adolescents with current voice-hearing experiences to a clinical comparison group without these experiences. All participants were recruited from secondary mental health services, and they were between 14–18 years of age. Guided by the literature about the voice-hearing prevalence in a wide range of diagnostic groups in youth [35], the focus was transdiagnostic. We hypothesised that young people with voice-hearing experiences, versus the clinical comparison group, would present with more social relating difficulties, measured by negative relating style, social comparison, perceived belongingness and connectedness, perceived strain, and support (hypothesis 1). With respect to potential explanatory factors, we hypothesised that young people with voice-hearing experiences would report higher levels of negative schemas, lower levels of positive schemas and higher current general psychopathology, specifically depression and anxiety (hypothesis 2), and higher levels of childhood trauma and poorer premorbid adjustment (hypothesis 3). In this study, schemas were defined as the generalised appraisals young people hold about themselves and other people, as they can influence their adaptation to the social world [36].

## Methods

### Design

The present study involved a cross-sectional design using a clinical comparison group, examining historical and concurrent associations with voice-hearing.

### Ethics approval

This study was performed in line with the principles of the Declaration of Helsinki. Approval was granted by the London—Brighton & Sussex Research Ethics Committee (reference number: 17/LO/2078).

### Participants

Sixty-eight participants were recruited from Child and Adolescent Mental Health Services (CAMHS) and Early Intervention in Psychosis (EIP) services within one NHS Trust between March 2018 and June 2019 (Fig 1 in S1 Appendix). Inclusion criteria for all participants were: 1) aged 14–18 years at referral and 2) capacity to provide written, informed consent. Exclusion criteria were: 1) insufficient English language ability, 2) moderate or severe learning disability and 3) immediate risk to self or others. For the voice-hearing group additional inclusion criteria were: 1) presence of voices for at least 3 months and 2) presence of voices within the past week. Exclusion criteria specific to this group were: 1) voice-hearing attributed to an organic illness or acute intoxication, solely to drug use or hypnagogic/ hypnopompic experiences or 2) voice-hearing of little clinical significance, e.g., one’s name being called, noises. The inclusion criterion specific to clinical comparison group was that young people did not report any current voice-hearing.

Recruitment took place via referrals from clinical teams or via parental or self-referrals. Written informed consent was provided by all participants and from a person with parental responsibility where the participant was under 16 years of age. The research assessment data were collected in multiple assessment meetings, spread over two or more sessions depending on participant’s preference and needs. All data were collected within one calendar month from the first research appointment. To convenience participants, the researcher could meet the young person at their place of residence, at their college, at their closest NHS service, at their GP surgery or at the local university. Authors had access to information that could identify individual participants during or after data collection.

### Measures

#### Sample demographic and clinical characteristics

Participants were asked to complete questions on demographic characteristics, diagnosis, and current treatment. To gather information on the clinical characteristics of the sample, the measures below were administered. The Comprehensive Assessment of At-Risk Mental States–Short form (CAARMS) [37] semi-structured interview was used to determine the presence of ultra-high-risk for psychosis or a first episode of psychosis status. An overall CAARMS symptom severity score was calculated as the summed scores of the product of severity and frequency ratings of the three symptom subscales, excluding perceptual abnormalities [38]. The Structured Clinical Interview for Axis-I DSM-IV Disorders (SCID-I-RV) [39] modules B (Psychotic symptoms) and C (Psychotic disorders) were rated for participants reaching the CAARMS psychosis threshold. The Mini International Neuropsychiatric Interview (MINI) for psychotic disorders studies (Version 7.0.2) [40] was completed to capture any research diagnosis for major psychiatric disorders. In the voice-hearing group, 20.59% heard voices at least once as week, 23.53% at least once a day, 11.76% at least once an hour and 44.12% continuously or almost continuously. Further details on the voice-hearing characteristics in the voice-hearing group can be found in another report [10].

#### Social relating

The Social Connectedness Scale (mSCS; as revised by Lee et al. [41]) captured sense of social connectedness and the Social Comparison Scale (SCS) [42] captured how individuals see themselves compared to social others in terms of social rank, attractiveness and belongingness. The shortened Person’s Relating to Others Questionnaire (PROQ-3) [43] was used to capture negative relating to others. Each subscale represented a negative state of relatedness, as reflected on two intersecting axes, with four poles: one of closeness (seeking to be involved) versus distance (withdrawing from others) and one of ‘upperness’ (relating from a position of dominance) versus ‘lowerness’ (relating from a position of submission). This creates the following subscale names: upper neutral (UN), upper close (UC), neutral close (NC), lower close (LC), lower neutral (LN), lower distant (LD), neutral distant (ND) and upper distant (UD). A score for each subscale and an overall negative relating score were calculated. The Support and Strain Scales self-report questionnaire (SSS) [44] was used as a measure of perceived support and strain from peers, family and romantic partners. For those without a romantic partner, mean overall support and strain was calculated from the peer and family scales only.

#### Putative explanatory factors

The Brief Core Schema Scales (BCSS) [36] self-report questionnaire was used to measure the level of negative and positive schematic beliefs about self and others, consisting of four subscales, namely “Positive Self”, “Positive Others”, “Negative Self” and “Negative Others”. Total scores were calculated for each subscale. The Childhood Trauma Questionnaire–Short Form (CTQ-SF) [45, 46] was administered to capture negative childhood experiences up to the age of participation, consisting of five subscales representing different types of trauma: physical, sexual and emotional abuse, emotional and physical neglect. Scores across all five subscales were totalled to create an overall score. The Premorbid Adjustment Scale (PAS) [47] semi-structured interview has been designed to capture retrospectively the level of functioning up to one year before the onset of psychosis in terms of developmental goals [48]. Due to the transdiagnostic study sample, all types of mental illness were considered. The Beck Depression Inventory-II (BDI-II) [49] and the Beck Anxiety Inventory (BAI) [50] were used to capture the presence and severity of current depressive and anxiety symptoms. Structured activity (SA) in the past month was calculated using the Time Use Survey interview (TUS; as adapted by Fowler and colleagues [51] from the UK 2000 Time Use Survey) [52] and it was considered an estimate of social and occupational functioning over the past month. Neurocognitive performance was captured in order to control for its effects, considering the it can negatively impact young people’s social functioning [53]. The Wechsler Memory Scale–third edition logical memory I (LM-I) interview test (WMS- III) [54] was used as a measure of auditory immediate memory giving a total immediate recall score. Executive function was assessed with the Controlled Word Association Test (COWAT) [55]. An overall neurocognitive performance score was calculated as the mean z-score from LM-I and COWAT.

### Statistical analyses

All analyses were carried out using SPSS for Windows, Version 25 [56]. Appropriate transformations were applied any outcome variables that deviated from normal distribution (S2 Appendix). To investigate between-group differences in demographic and clinical characteristics independent sample t-tests, Mann-Whitney U tests and likelihood ratio chi-square tests were conducted before the main analysis (Bonferroni-corrected critical p-value = .002).

Missing data were evaluated using missing values analysis to identify any patterns in the missingness of data. Independent sample t-tests, Mann-Whitney U tests and Fisher’s exact tests were carried out to investigate whether missingness was related to any demographic or clinical presentation variables comparing those who did and did not complete the study measures within each study group. Where possible, bias-corrected and accelerated bootstrap intervals (BCa95%CI) using 2000 samples were calculated to ensure the robustness of the results due to the small size of the compared groups. A Bonferroni-corrected p-value of .001 accounted for multiple comparisons.

Analyses of covariance (ANCOVAs) were used to assess between-group differences on social relating variables, namely, the negative relating sub-scales and overall negative relating to others (PROQ-3), social comparison ratings (SCS), perceived belongingness (SCS belongingness subscale), social connectedness (mSCS) and social support and strain (SSS). Age, current functioning (Time Use SA), depression (BDI-II), anxiety (BAI), current psychotic psychopathology (CAARMS overall severity) and overall neurocognitive functioning performance (mean z-score from LM-I and COWAT) were considered conceptual covariates (hypothesis 1). The significance value was corrected for multiple testing (Bonferroni-corrected p = .002). Two ANCOVAs were carried out to compare the groups on schemas about self and others (BCSS), while adjusting for age, current functioning (Time Use SA) and overall neurocognitive functioning performance (Bonferroni-corrected p-value = .008), and to assess between-group differences on depression (BDI-II) and anxiety (BAI), while adjusting for age and neurocognitive performance (hypothesis 2) (Bonferroni-corrected p-value = .01). ANCOVAs were also performed to assess between-group differences on childhood trauma (Overall CTQ) and premorbid adjustment (mean overall PAS) while adjusting for age (hypothesis 3) (Bonferroni-corrected p-value = .01).

ANCOVA assumptions testing indicated that the levels of BDI-II, BAI, current CAARMS overall severity differed significantly between the two groups, violating the assumption of independence of covariate and predictor effect. Thus, these variables were excluded from the analysis testing for hypothesis 1.

## Results

### Missing data

Missing values analysis indicated that the highest rate of missing cases was 17.6% for childhood trauma in the voice-hearing group and 11.8% for the same variable as well as for overall negative relating and anxiety scores in the comparison group (Table 1 in S3 Appendix). In the voice-hearing group, Mann-Whitney U, independent samples t- tests and Fisher’s exact tests did not find any differences between the completers and non-completers of any variables, *p*s > .001 (Bonferroni corrected critical p-value). In the comparison group, Fisher’s exact test indicated a significant difference in the presence of ever having received psychological therapy in completers (29/29) compared to non-completers (0/3) of the SSS Overall Strain Scale, *p* < .001. Analysis was carried out using all available cases.

### Sample characteristics

Table 1 presents the demographic characteristics of the sample. In total, N = 55 young people were recruited from CAMHS and N = 13 from EIP services. Details on the clinical characteristics of the two study groups can be found in Table 1 in S4 Appendix.

**Table 1:** Sample characteristics for the voice-hearing (N = 34) and clinical comparison (N = 34) groups.

Sample characteristic	Voice-hearers (N = 34)	Comparison group (N = 34)
	**M (Min-Max; SD)**	
**Age**	16.28 (14–18.95; 1.09)	16.59 (14.03–18.44; 1.26)
	**N (Valid %)**	
**Gender**		
Male	7 (20.59)	9 (26.47)
Female	25 (73.53)	25 (73.53)
Another gender identity	2 (5.88)	0
**Ethnicity**		
White British	29 (85.29)	32 (94.12)
Another ethnicity	5 (14.71)	2 (5.88)
**Accommodation type** ^**a**^		
Owner occupied	15 (45.45)	21 (61.76)
Rented (Privately)	8 (24.24)	8 (23.53)
Rented (Local authority)	9 (27.27)	4 (11.76)
Other type of accommodation	1 (3.03)	1 (2.94)
**Educational level**		
None	22 (64.71)	14 (41.18)
GCSEs or equivalent	9 (26.47)	18 (52.94)
A level or equivalent	3 (8.82)	2 (5.88)
**Limited day-to-day activities due to disability**		
A lot	1 (2.94)	2 (5.88)
A little	5 (14.71)	5 (14.71)
No	28 (82.35)	26 (76.47)
Prefer not to say	0	1 (2.94)
**Student/Employment status** ^**b**^		
Student	31 (91.18)	24 (70.59)
Employed part time (paid)	9 (26.47)	11 (32.35)
Employed part time (voluntary)	0	1 (2.94)
Unemployed (receiving state benefits)	1 (2.94)	1 (2.94)
Unemployed (not receiving state benefits)	0	6 (17.65)
**Had a self-reported mental health diagnosis**	24 (70.59)	25 (73.53)
**Self-reported diagnosis of psychosis**	5 (14.71)	2 (5.88)
**Were taking any medication for mental health problems**	24 (70.59)	17 (50)
**Had received any psychological therapy**	31 (91.18)	31 (91.18)
**Type of mental health service**		
Child and Adolescent Mental Health	28 (82.35)	27 (79.41)
Early Intervention in Psychosis	6 (17.65)	7 (20.59)

All frequency and descriptive statistics are reported on raw untransformed data; *M* = Mean; *SD* = standard deviation; Valid % represents percentage of participants with available data.

^a^ *N* = 1 missing from the voice-hearing group

^b^ multiple responses allowed

Regarding demographic and clinical characteristics, the two study groups significantly differed in the severity of current psychotic symptomatology (excluding perceptual abnormalities) with the clinical comparison group having significantly lower scores (M = 23.53, SD = 17.72, 95%CIs, [17.24, 29.71]) compared to voice-hearers (M = 39.03, SD = 19.50, 95%CIs [31.75, 46.31]), *t* (62) = -1.55, *p* = .001, *d* = .87. No other statistically significant between-group difference was found (Tables 2 and 3 in S4 Appendix). In the voice-hearing group, 31 young people scored over psychosis threshold on CAARMS however, only three met these criteria for psychotic symptoms other than voice-hearing. In terms of voice-hearing, 20.59% heard voices at least once a week, 23.53% at least once a day, 11.76% at least once an hour and 44.12% continuously or almost continuously. Further details on the voice-hearing characteristics in the voice-hearing group can be found in another report [10].

### Between-group differences in social relating (hypothesis one)

Table 2 summarises the results of the one-way ANOVAs and ANCOVAs used to identify between-group differences in social relating variables between voice-hearers and the clinical comparison group, firstly without adjusting for any covariates and then adjusting for all covariates simultaneously.

**Table 2:** ANOVA and ANCOVA results for social relating variables comparing the voice-hearing group (N = 34) to the comparison group (N = 34).

	ANOVA			ANCOVA adjusting for all covariates			
Outcome variable	F (df_1_,df_2_)	Partial η^2^	p	F (df_1_,df_2_)	Partial η^2^	Adj R^2^	p
Upper neutral (UN)	0.0004 (1,64)	.000006	.985	0.208 (1,60)	.003	.011	.650
Upper close (UC)	1.02 (1,64)	.016	.316	0.873 (1,60)	.014	.009	.354
Neutral close (NC)	2.04 (1,64)	.031	.158	1.406 (1,60)	.023	.037	.240
Lower close (LC)	7.51 (1,64)	.105	.008	7.77 (1,60)	.115	.11	.007
Lower neutral (LN)	.376 (1,64)	.006	.542	0.15 (1,60)	.003	.074	.699
Lower distant (LD)	2.011 (1.63)	.031	.161	1.62 (1,59)	.027	.04	.209
Neutral distant (ND)	5.74 (1,63)	.084	.02	5.29 (1,59)	.082	.04	.025
Upper distant (UD)	.010 (1,62)	.0002	.92	0.043 (1,58)	.001	-.048	.837
Overall PROQ-3^a^	5.11 (1,61)	.077	.027	4.99 (1,59)	.078	.032	.029
Social comparison subscale of Belongingness^b^	1.76 (1,64)	.027	.189	1.41 (1,61)	.023	-.025	.239
Sum of Social Comparison Scale	5.76 (1,64)	.083	.019	4.64 (1,61)	.071	.028	.035
Total Social Connectedness Scale^a^	3.47 (1, 61)	.054	.067	6.42 (1,59)	.098	.138	.014
Mean Family support	2.37 (1,65)	.035	.128	3.99 (1,61)	.061	.06	.050
Mean Partner support	1.38 (1,28)	.047	.251	0.46 (1,25)	.018	.113	.506
Mean Friends support	3.18 (1,64)	.047	.08	5.29 (1,60)	.081	.182	.025
Mean Family strain	1.5 (1,61)	.024	.225	2.80 (1,57)	.047	.016	.100
Mean Friends strain	1.46 (1,61)	.023	.231	0.38 (1,59)	.006	.091	.538
Mean Partner strain	1.23 (1,28)	.042	.277	1.28 (1,25)	.049	-.025	.269
Overall Support^b^	3.29 (1,64)	.049	.074	3.14 (1,61)	.049	.01	.082
Overall Strain	1.21 (1,60)	.003	.652	0.54 (1,56)	.01	.068	.466

df_1_ = degrees of freedom for the effect of the model; df_2_ = degrees of freedom for the residual; Partial η^2^ = partial eta-squared; Adj R^2^ = Adjusted R-squared; PROQ-3 = Shortened Person’s Relating to Others Questionnaire; Values presented for the UN and ND PROQ-3 subscales, Mean Family support, Mean Partner support, Mean Friends support, Mean Friends strain and Mean Partner strain are based on the transformed variables. Values in bold font highlight statistically significant effects.

^a^ Neurocognitive performance did not meet ANCOVA assumptions and was excluded from the final ANCOVA

^b^ a not meet ANCOVA assumptions and was excluded from the final ANCOVA

After controlling for all covariates, one-way ANCOVAs showed a significant main association between being a voice-hearer and lower close (LC), neutral distant (ND), and overall negative relating (Overall PROQ-3), with voice-hearers relating more negatively to others (M = 11.97, SD = 3.97; M = 10.18, SD = 4.26; M = 65.82, SD = 17 respectively) compared to the non-voice-hearing group (M = 9.42, SD = 3.57; M = 2.49, SD = .80; M = 8.16, SD = 3.48; M = 56.43, SD = 15.85, respectively). Social comparison scores were also found to be significantly lower in voice-hearers (M = 39.03, SD = 18.51) compared to the non-voice-hearing group (M = 49.32, SD = 16.31), indicating that voice-hearers tend to consider themselves of lower social rank compared to social others.

Voice-hearers additionally reported significantly lower perceived social connectedness (M = 21.90, SD = 10.56) compared to the non-voice-hearing group (M = 27.03, SD = 11.23). However, this association only became significant when adjusting for the effect of age, with being a voice-hearer explaining 10% (*p* = .014), age explaining 13% (*p* = .004), and current functioning .5% (*p* > .05) of the variance in social connectedness unattributable to other variables in the analysis. Social connectedness correlated significantly with age within the comparison group, *r*_*s*_ = -.51, *p* = .002, N = 33, in contrast to the voice-hearing group, *r* = -.16, *p* = .386, N = 30.

Additionally, the final ANCOVA model indicated that voice-hearers reported receiving lower support from their peers (M = 2.44, SD = .91) compared to young people without voice-hearing experiences (M = 2.83, SD = .93). Nevertheless, a significant difference between the two groups in mean friends support scores was found only when age was covaried; with group membership explaining 8% (*p* = .025), age 19% (*p* < .001), current functioning 1.4% (*p* > .05) and neurocognitive performance .9% (*p* > .05) of the variance in the participants scores that are not attributed to other variables in the analysis. Controlling for the effect of age showed that mean friends support correlated significantly with age within the comparison group, *r*_*s*_ = -.58, *p* < .001, N = 33, in contrast to the voice-hearing group, *r*_*s*_ = -.21, *p* = .250, N = 33. All associations were non-significant under the Bonferroni-corrected alpha level (*p* = or < .002).

### Between-group differences in interpersonal schematic beliefs and clinical symptomatology (hypothesis two)

Table 3 presents the results of the one-way ANOVAs and ANCOVAs used to identify between-group differences in BCSS schematic beliefs and in BDI-II depression scores and BAI anxiety scores, firstly without adjusting for any covariates and then adjusting all covariates at the same time.

**Table 3:** ANOVA and ANCOVA results for interpersonal schematic beliefs and current clinical symptomatology comparing the voice-hearing group (N = 34) to the comparison group (N = 34).

	ANOVA			ANCOVA adjusting for all covariates			
Outcome variable	F (df_1_,df_2_)	Partial η^2^	p	F (df_1_,df_2_)	Partial η^2^	Adj R^2^	p
Negative Self BCSS total	11.97(1,64)	.158	.001	11.67 (1,60)	.163	.122	.001
Positive Self BCSS total	6.27 (1,64)	.089	.015	4.83 (1,60)	.075	.053	.032
Negative Other BCSS total	13.13 (1,60)	.18	.001	10.05 (1,56)	.152	.124	.002
Positive Other BCSS^a^	4.53 (1,62)	.068	.037	5.16 (1,59)	.08	.048	.027
BDI-II total	16.22 (1,61)	.21	< .001	16.86 (1, 57)	.23	.20	< .001
BAI total^b^	20.90 (1,62)	.25	< .001	19.63 (1,61)	.24	.23	< .001

df_1_ = degrees of freedom for the effect of the model; df_2_ = degrees of freedom for the residual; Partial η^2^ = partial eta-squared; Adj R^2^ = Adjusted R-squared; BCSS = Brief Core Schema Scales; BDI- II = Beck Depression Inventory-II, BAI = Beck Anxiety Inventory. Values presented for BAI total are based on the transformed variable. Values in bold font highlight statistically significant effects.

^a^Age did not meet ANCOVA assumptions and was excluded from the final ANCOVA

^b^Neurocognitive performance did not meet ANCOVA assumptions and was excluded from the final ANCOVA.

Voice-hearers scored significantly higher in negative self-beliefs (M = 12.97, SD = 6.45) compared to the non-voice-hearing group (M = 7.94, SD = 5.33). A similar main effect was found for negative beliefs about others, with voice-hearers endorsing greater negative other schematic beliefs (M = 12.58, SD = 5.46) when compared to the non-voice-hearing group (M = 7.68, SD = 5.19). Additionally, the voice-hearing group scored significantly lower in positive self-beliefs (M = 6.5, SD = 4.72) compared to the non-voice-hearing group (M = 9.79, SD = 5.86), as well as in positive other beliefs (M = 8.22, SD = 3.79) compared to the non-voice-hearing group (M = 10.66, SD = 5.25). After considering the Bonferroni corrected significance threshold (*p* = or < .002), the between group differences in negative self and in negative other belief scores were the only associations that remained statistically significant.

Regarding clinical symptomatology, after adjusting for age and neurocognitive performance, belonging to the voice-hearing group had a significant association with BDI-II total scores, with voice-hearers reporting greater depression levels (M = 38.13, SD = 11.58) compared to the comparison group (M = 24.42, SD = 15.02). A one-way ANCOVA adjusted for the effect of age also showed that voice hearers reported higher anxiety levels (M = 33.59, SD = 14.31) compared to the comparison group (M = 17.93, SD = 13.92).

### Between-group differences in premorbid adjustment and childhood trauma (hypothesis three)

Table 4 presents the results of the one-way ANOVAs and ANCOVAs used to identify between-group differences in childhood trauma scores (Overall CTQ), premorbid adjustment (Mean overall PAS) and in clinical symptomatology, namely BDI-II depression scores and BAI anxiety scores, without adjusting for any covariates and then adjusting for all covariates at the same time.

**Table 4:** ANOVA and ANCOVA results for overall childhood trauma and premorbid adjustment comparing the voice-hearing group (N = 34) to the comparison group (N = 34).

	ANOVA			ANCOVA adjusting for all covariates			
Outcome variable	F (df_1_,df_2_)	Partial η^2^	p	F (df_1_,df_2_)	Partial η^2^	Adj R^2^	p
Mean Overall PAS	14.02 (1,64)	.18	< .001	7.4 (1,63)	.19	.17	< .001
Overall CTQ	.522 (1,56)	.01	0.473	1.23 (1,55)	.02	.08	.273

df_1_ = degrees of freedom for the effect of the model; df_2_ = degrees of freedom for the residual; Partial η^2^ = partial eta-squared; Adj R^2^ = Adjusted R-squared; PAS = Premorbid Adjustment Scale; CTQ = short-form Childhood Trauma Questionnaire; Values presented for Overall CTQ are based on the transformed variable. ANCOVAs for Mean Overall PAS and Overall CTQ were adjusted only for Age. Value in bold font highlights statistically significant effects.

A one-way ANCOVA adjusted for the effect of age showed that voice-hearers had worse mean overall premorbid adjustment (PAS) (M = .38, SD = .14) compared to the comparison group (M = .26, SD = .10). No between-group differences were identified in the overall CTQ trauma scores (*p* < .05), with both groups having a median of 40 (voice-hearing group, M = 47.6; SD = 20.21; comparison group, M = 43.4; SD = 16.75).

## Discussion

Our findings suggest that voice-hearing in young people who experience mental health difficulties could be an index for social relating difficulties and more severe concurrent psychopathology. The voice-hearing group reported relating to others more negatively overall, using more lower close and neutral distant patterns of negative relating, perceived themselves of lower social rank compared to social others, reported receiving lower support from their friends and feeling less socially connected with the world and others. These correlations were not, however, robust to a correction for multiple testing. Potential explanatory factors were explored. The two groups did not differ significantly in terms of overall childhood trauma levels, however the voice-hearing group scored significantly lower on overall premorbid adjustment and higher on negative self and other schematic beliefs, depressive and anxiety symptoms as well as on the severity of current PE, other than voice-hearing.

### Social relating difficulties and voice-hearing in youth

Higher scores on lower close and neutral distant relating in the voice-hearing group indicate a tendency to relate in two seemingly contrasting ways; being afraid of abandonment and constantly seeking reassurance and acceptance and also, being uncomfortable when others are too close, avoiding social activities, keeping to oneself and pushing others away [57, 58]. This might express an oscillation between wanting to be supported by others while fearing of losing them and thus, distancing themselves to either avoid abandonment or rejection. Potentially, both relating styles could be linked with fear of being identified as voice-hearers and endangerment of their social status in peer groups and subsequent abandonment [59]. A tendency to keep others away could also be an effort to avoid stigmatizing responses [60], communication with others feeling too overwhelming if voice-hearing becomes disruptive [15] or because they might engage with voices instead of non-voice others, with voices fulfilling the role of social relationships [12].

Compared to the clinical control group, voice-hearers reported feeling less socially connected and receiving less support from friends, which aligns with existing evidence linking at-risk for psychosis status in youth [3, 4]. Considering the important role of friends’ support in adolescence [61], lower perceived social support means there may be little to buffer against stress and there may be higher vulnerability to experiencing voice-hearing [62]. Alternatively, voice-hearing onset may interrupt friend support [63] as a function of young voice-hearers using less approach-oriented coping [4, 33], not disclosing their voice-hearing to peers for stigma-related reasons and/or due to lack of interpersonal trust [14, 59]; thus providing friends with fewer opportunities to provide support. Additionally, feeling socially disconnected from others in daily life could result from the voices eliciting feelings of stigma, fear, and loneliness [15]. This in turn, could make young people more likely to engage with the voices, limit their interactions with others, thus diminishing their sense of connectedness.

### Potential explanatory factors

The current study provided some evidence that psychological and psychopathological factors may partially explain social relating difficulties in youth voice-hearing. Young people with voice-hearing experiences reported holding both self and other negative schematic beliefs with more conviction and a tendency to hold positive schematic beliefs less strongly. This is in agreement with findings from a clinical sample of children and adolescents which found voice severity to be negatively correlated with positive self-beliefs [27]. Further supporting the link between negative schemas and PE experiences in youth, in a transdiagnostic sample of young people referred to CAMHS, negative schematic beliefs about the self were found to be significantly implicated in hallucination severity and overall schemas were the principal psychological factor explaining overall PE severity [64]. Young people with voice-hearing experiences also reported poorer premorbid adjustment, which could partially reflect the negative early life interpersonal experiences that contributed to the formation of negative schematic beliefs [65]. Poorer premorbid adjustment could also be a marker for social relating issues that start before the onset of voice-hearing [22], and which could further deteriorate at and after the onset of voice-hearing [7]. Contrary to initial hypothesis, the study groups did not differ in terms of overall childhood trauma levels. However, childhood trauma prevalence was high in both groups; potentially indicating that underlying trauma is not linked with voice-hearing specifically [66] or perhaps only with its severity and not its presence [67, 68]. It is important to acknowledge that the trauma measure used did not capture common traumatic experiences that may be related to voice-hearing, such as emotional bullying outside the family [68].

In terms of co-occurring psychopathology, the voice-hearing group scored higher on measures of anxiety and depressive symptoms as well as other PE compared to the clinical comparison group. This was in accordance with youth population-based research associating the presence of voice-hearing with a wide range of psychopathologies [31], including symptoms of depression, anxiety [69, 70] and other PE [71]. Furthermore, persistent or distressing PE in youth have been linked with elevated levels of depression and anxiety both cross-sectionally and longitudinally [20, 72]. Thus, the present findings could support the premise that voice-hearing might be an index of a more complex and severe clinical presentation which could be contributing to social relating difficulties [18, 31]. Specifically, evidence shows that youth with PE, including voice-hearing, have an increased risk for multi-morbid lifetime psychiatric disorders as well as poorer current social and global functioning when compared to youth who did not report PE [69, 73].

Voice-hearing could also be a marker for a complex clinical presentation that crosses several diagnostic domains [74] and thus, be linked to lower social functioning. This would be in agreement with the literature suggesting that comorbidity is a marker of severity [75] and specifically, that co-occurrence of PE with diagnosable psychiatric diagnoses in early adolescence constitutes a risk factor for later mental health diagnoses and treatment [76]. Based on previous research, emotional difficulties might explain a considerable part of the relationship between PE and social functioning in adolescence [18]. This could be either by contributing to negative appraisals of voices, potentially eliciting further increase in depression, anxiety and maintenance of voice-hearing [77] or as part of the pathway from voice-hearing to poor social functioning, as young people might develop low mood and anxiety due to their unusual perceptual experiences [77]. These two affective domains of symptomatology could also contribute to social withdrawal [78] and therefore directly interfere with social relating. Based on this explanation, voice-hearing might not relate directly or significantly with some social relating outcomes when other psychopathology is considered. In addition, voice-hearing could have uniquely negatively influenced young people’s social relating. Indirect influences could include social distancing due to fear of being stigmatised [7] or undermining trust in others via their content [14, 15] while direct influences could revolve around voice-hearing occurrence interrupting social interactions or interfering with young people’s concentration [13, 15], leading to social withdrawal [7]. The unique connection of voice-hearing with social relating has also been supported in clinical studies where the presence of PE in adolescents with non-psychotic disorders predicts social functioning problems over and above multimorbidity [32–34].

### Limitations

Several limitations to the current study should be acknowledged. First, its cross-sectional design precludes causal inference and thus, the issue of temporality between voice-hearing, social relating variables, and co-occurring psychopathology could not be addressed. Second, the low prevalence of voice-hearing [79] and the time restrictions of this research, led to a small sample size and thus, negative results should be interpreted with caution as they might not represent a true absence of effects. Third, one-to-one matching between the two groups based on diagnosis was not performed, thus not allowing for more confident inferences with regards to the unique associations between voice-hearing and social relating, by accounting for underlying psychopathology effects. Nevertheless, there were no significant differences between the two groups in terms of non-psychotic research diagnoses. Fourth, there was a gender imbalance within both study groups, with 74% in both groups identifying as females. Female adolescents might be more likely to report their distress [80] or more likely to seek help when distressed [81]. An additional explanation could be that young people in the present research had a clinical profile similar to that found in [82] who noted that young people with voice-hearing and a diagnosis of Borderline Personality Disorder (BPD) are more likely to be female compared to young people with a diagnosis of a schizophrenia spectrum disorder and voice-hearing. This is in accordance with gender differences observed in clinical settings [83, 84]. Nevertheless, the study did not assess for the presence of BPD characteristics and cannot clarify this. Finally, this study did not control negative symptoms which have been implicated in functioning difficulties in youth samples at risk for psychosis with positive psychotic experiences [85]. Future research could investigate the role of negative symptoms in young voice-hearer’s social relationships, to further clarify whether voice-hearing is linked to social relating difficulties independently of associations with problems with motivation and interest and expressive functions [86].

### Clinical implications

Voice-hearing in help-seeking youth could be a marker for current social relating problems as well as for co-occurring psychopathology, including depression, anxiety, and other psychotic symptoms. As voice-hearing has been linked with poorer mental health, and social and global functional outcomes that seem to persist throughout adolescence to early adulthood [63, 73], young people who report such symptoms could be considered a target group for interventions to reduce distress, ameliorate current difficulties with social relating and potentially reduce the likelihood of poor long-term outcomes. Given the social relating profile of the young people with voice-hearing experiences, relating-based therapies for voices similar to those developed for adults might be helpful (e.g., Relating Therapy) [87]. This type of therapy focuses on developing adaptive ways of responding to difficult relationships with others, regardless of whether this is people in the individual’s environment (e.g., with peers) or voices [10]. A therapeutic focus on negative schematic beliefs could also be an important treatment target, while considering their connection with low mood and anxiety which could be contributing to voice-hearing severity as well as vice versa. A potentially beneficial therapy could be cognitive behavioural therapy for voices, which is currently being evaluated in young people, in a digital self-help form [88].

### Conclusions

Although poorer premorbid adjustment might indicate an early vulnerability to social relating difficulties, voice-hearing in help-seeking youth could be a marker for social relating problems. Voice-hearing also seems to co-occur with other mental health difficulties, including depression, anxiety, and other psychotic symptoms, extending prior evidence of voice-hearing being a risk marker for increased psychopathological complexity. Therapy approaches for voice-hearing in youth that take into account social relating difficulties, negative schematic beliefs, and co-occurring psychopathology could be beneficial.

## Supporting information

S1 Checklist STROBE statement—checklist of items that should be included in reports of observational studies. (DOCX)

S1 Appendix Recruitment information. (DOCX)

S2 Appendix Additional preliminary analysis information. (DOCX)

S3 Appendix Missing data information. (DOCX)

S4 Appendix Additional tables of results. (DOCX)
