# Supplementary material for: What’s the impact of voice-hearing experiences on the social relating of young people: A comparison between help-seeking young people who did and did not hear voices
Source: PLoS One. 2023 Sep 26;18(9):e0290641. doi: 10.1371/journal.pone.0290641 (PMC10522017; doi:10.1371/journal.pone.0290641)
Supplement: S1 Appendix — (DOCX) [file pone.0290641.s002.docx]

## S1 Appendix. Recruitment information

Fig 1. Vista Project Recruitment Flow Diagram. LP = Lead practitioner; CAMHS = Child and Adolescent Mental Health Services; EIP = Early Intervention in Psychosis services.

71 participants consented to take part in the Vista study. 4 participants did not continue with the study after the first meeting with the researcher. Data from these 4 participants were not included in any subsequent analysis. From the remaining 68 participants, 34 participants met the criteria for the voice-hearing group and 34 were allocated to the clinical comparison group.

From the 34 in the voice-hearing group, 4 participants did not complete the whole study assessment. All 4 participants met with the researcher twice and completed an average of 3.73 hours of assessment time completing a big part of the assessment measures.

Reasons for missing data from the 4 cases that did not complete the full assessment were the following: issues with speech (n = 1), no longer wished to continue (n = 1), lead practitioner requested to pause participation following concerns about risk (n = 1) and then participant disengaged or disengaged following a holiday period (n = 1). Reasons for other missing values in the data were not recorded.
