# Supplementary material for: What’s the impact of voice-hearing experiences on the social relating of young people: A comparison between help-seeking young people who did and did not hear voices
Source: PLoS One. 2023 Sep 26;18(9):e0290641. doi: 10.1371/journal.pone.0290641 (PMC10522017; doi:10.1371/journal.pone.0290641)
Supplement: S2 Appendix — (DOCX) [file pone.0290641.s003.docx]

## **S2 Appendix.** Additional preliminary analysis information

To minimise bias due to normality issues, a square root transformation was used to correct the upper neutral (UN) negative relating subscale, the mean Friends Strain subscale, current CAARMS Overall severity, and BAI variables. A log10 transformation was applied to the mean Partner Strain subscale, Overall CTQ and a reverse square root transformation was used to correct the neutral distant (ND) negative relating PROQ-3 subscale. A reverse square root transformation adding a constant score of 1 was used for the Mean Friends Support and Mean Family Support subscales. Lastly, a reciprocal reverse transformation adding a constant of 1 was used for the Mean Partner Support subscale.
