# Supplementary material for: What’s the impact of voice-hearing experiences on the social relating of young people: A comparison between help-seeking young people who did and did not hear voices
Source: PLoS One. 2023 Sep 26;18(9):e0290641. doi: 10.1371/journal.pone.0290641 (PMC10522017; doi:10.1371/journal.pone.0290641)
Supplement: S3 Appendix — (DOCX) [file pone.0290641.s004.docx]

## S3 Appendix. Missing data information

Table 1. Missingness rates for main study variables for voice-hearers (N =34) and the comparison group (N=34).

|  | **Voice-hearers (N = 34)** | | | | **Comparison group (N =34)** | | | | | |
| --- | --- | --- | --- | --- | --- | --- | --- | --- | --- | --- |
| Sample characteristic | Overall Missing | | Not administered | | Overall Missing | | | Not administered | | |
|  | N | % | N | % | N | % | | N | % | |
| **BCSS** |  |  |  |  |  |  | |  |  | |
| Negative Self beliefs | 2 | 5.9 | 1 | 2.9 | 0 | 0 | | 0 | 0 | |
| Positive Self beliefs | 2 | 5.9 | 1 | 2.9 | 0 | 0 | | 0 | 0 | |
| Negative Other beliefs | 3 | 8.8 | 1 | 2.9 | 3 | 8.8 | | 0 | 0 | |
| Positive Other beliefs | 2 | 5.9 | 1 | 2.9 | 2 | 5.9 | | 0 | 0 | |
| **PROQ-3** |  |  |  |  |  |  | |  |  | |
| PROQ-3 – UN | 1 | 2.9 | 1 | 2.9 | 1 | 2.9 | | 0 | 0 | |
| PROQ-3 – UC | 1 | 2.9 | 1 | 2.9 | 1 | 2.9 | | 0 | 0 | |
| PROQ-3 – NC | 1 | 2.9 | 1 | 2.9 | 1 | 2.9 | | 0 | 0 | |
| PROQ-3 – LC | 1 | 2.9 | 1 | 2.9 | 1 | 2.9 | | 0 | 0 | |
| PROQ-3 – LN | 1 | 2.9 | 1 | 2.9 | 1 | 2.9 | | 0 | 0 | |
| PROQ-3 – LD | 1 | 2.9 | 1 | 2.9 | 2 | 5.9 | | 0 | 0 | |
| PROQ-3 – ND | 1 | 2.9 | 1 | 2.9 | 2 | 5.9 | | 0 | 0 | |
| PROQ-3 – UD | 1 | 2.9 | 1 | 2.9 | 3 | 8.8 | | 0 | 0 | |
| PROQ-3 Overall Negative relating | 1 | 2.9 | 1 | 2.9 | 4 | 11.8 | | 0 | 0 | |
| SCS Belongingness | 2 | 5.9 | 2 | 5.9 | 0 | | 0 | 0 | | 0 |
| SCS Total | 2 | 5.9 | 2 | 5.9 | 0 | | 0 | 0 | | 0 |
| mSCS Total | 4 | 11.8 | 2 | 5.9 | 1 | | 2.9 | 0 | | 0 |
| SSS Family Support | 1 | 2.9 | 1 | 2.9 | 0 | | 0 | 0 | | 0 |
| SSS Friends Support | 1 | 2.9 | 1 | 2.9 | 1 | | 2.9 | 0 | | 0 |
| SSS Partner Support (if applicable) | 2 | 5.9 | 1 | 2.9 | 0 | | 0 | 0 | | 0 |
| SSS Family Strain | 3 | 8.8 | 1 | 2.9 | 2 | | 5.9 | 0 | | 0 |
| SSS Friends Strain | 2 | 5.9 | 1 | 2.9 | 1 | | 2.9 | 0 | | 0 |
| SSS Partner Strain (if applicable) | 1 | 2.9 | 1 | 2.9 | 1 | | 2.9 | 0 | | 0 |
| SSS Mean overall Support | 1 | 2.9 | 1 | 2.9 | 1 | | 2.9 | 0 | | 0 |
| SSS Mean overall Strain | 3 | 8.8 | 1 | 2.9 | 3 | | 8.8 | 0 | | 0 |
| Mean Overall PAS | 2 | 5.9 | 2 | 5.9 | 0 | | 0 | 0 | | 0 |
| Overall CTQ | 6 | 17.6 | 1 | 2.9 | 4 | | 11.8 | 0 | | 0 |
| CAARMS overall severity^a^ | 4 | 11.8 | 4 | 11.8 | 0 | | 0 | 0 | | 0 |
| BDI-II total | 4 | 11.8 | 0 | 0 | 1 | | 2.9 | 0 | | 0 |
| BAI total | 0 | 0 | 0 | 0 | 4 | | 11.8 | 0 | | 0 |
| Overall neurocognitive performance | 2 | 5.9 | 2 | 5.9 | 0 | | 0 | 0 | | 0 |
| Note*.* BCSS= Brief Core Schema Scales; PROQ-3 = shortened Person's Relating to Others Questionnaire; SCS = Social Comparison Scale; mSCS = Social Connectedness Scale; SSS = Strain and Support Scales; SIM= Social Identity Mapping; PAS = Premorbid Adjustment Scale; CTQ = Childhood Trauma Questionnaire; Comprehensive Assessment of At-Risk Mental States – Short form; BDI-II= Beck’s Depression Inventory - II; BAI = Beck Anxiety Inventory  ^a^not administered at least one of the sections of the measure | | | | | | | | | | |
