# Supplementary material for: What’s the impact of voice-hearing experiences on the social relating of young people: A comparison between help-seeking young people who did and did not hear voices
Source: PLoS One. 2023 Sep 26;18(9):e0290641. doi: 10.1371/journal.pone.0290641 (PMC10522017; doi:10.1371/journal.pone.0290641)
Supplement: S4 Appendix — (DOCX) [file pone.0290641.s005.docx]

## **S4 Appendix. Additional tables of results**

Table 1. Frequency statistics for the clinical characteristics of the voice-hearing (N = 34) and clinical comparison (N = 34) groups.

| **Sample characteristic** | **N (Valid %)** | |
| --- | --- | --- |
|  | **Voice-hearers (N = 34)** | **Comparison group (N = 34)** |
| **UHR status (CAARMS)** |  |  |
| Not at-risk | 1 (2.94) | 15 (44.12) |
| At -risk | 2 (5.88) | 7 (20.58) |
| Over psychotic threshold | 31 (91.18) | 12 (35.29) |
| **SCID Psychotic disorders** |  |  |
| Not meeting criteria/Not applicable | 4 (11.76) | 29 (85.29) |
| Schizophrenia | 8 (23.53) | 1 (2.94) |
| Schizoaffective | 2 (5.88) | 0 |
| Schizophreniform | 0 | 1 (2.94) |
| Psychotic Mood Disorder | 0 | 1 (2.94) |
| Psychotic Disorder Not Otherwise Specified | 20 (58.82) | 2 (5.88) |
| **MINI diagnostic categories^a^** |  |  |
| MDE | 9 (28.13) | 7 (20.59) |
| Past MDE | 28 (87.50) | 30 (88.24) |
| Manic Episode | 0 | 1 (2.94) |
| Past Manic Episode | 9 (28.13) | 5 (14.71) |
| Past Hypomanic Episode | 1 (3.13) | 0 |
| Hypomanic Symptoms | 1 (3.13) | 0 |
| Past Hypomanic Symptoms | 0 | 1 (2.94) |
| Panic Disorder | 12 (38.71) | 6 (17.65) |
| Past Panic disorder | 19 (61.29) | 15 (44.12) |
| Agoraphobia | 9 (29.03) | 4 (11.76) |
| Social Anxiety | 19 (61.29) | 9 (26.47) |
| Obsessive compulsive disorder | 6 (19.35) | 6 (17.65) |
| Post-traumatic stress disorder | 7 (22.58) | 3 (9.09) |
| Alcohol Use disorder 12 months | 7 (22.58) | 5 (14.71) |
| Substance Use Disorder 12 months | 7 (22.58) | 12 (35.29) |
| Anorexia Nervosa (Restricting) | 1(3.23) | 1 (2.94) |
| Bulimia Nervosa | 4 (12.90) | 0 |
| Binge Eating | 0 | 3 (8.82) |
| Generalised Anxiety Disorder | 2 (6.45) | 7 (20.59) |
| Note. *M* = Mean; *SD* = standard deviation; Valid % represents percentage of participants with the available data; UHR = ultra high-risk for psychosis; SCID = Structured Clinical Interview for Axis-I DSM-IV Disorders; MINI = Mini International Neuropsychiatric Interview; MDE = Major Depressive Episode.  ^a^ From MDE to Past Hypomanic Symptoms, *N* = 2 missing from the voice-hearing group and *N* = 3 missing from the rest of MINI categories. *N* = 1 missing from the comparison group for PTSD.  All frequency statistics are reported on raw untransformed data. | | |

Table 2. Between-group differences in continuous demographic and clinical variables for the voice-hearing (N = 34) vs. comparison (N = 34) groups.

|  | **Voice-hearers (N = 34)** | | **Comparison group (N = 34)** | |  |  |
| --- | --- | --- | --- | --- | --- | --- |
|  | **M (Min- Max; SD)** | **BCa 95% CIs of Mean [LL, UL]** | **M (Min- Max; SD)** | **BCa 95% CIs of Mean [LL, UL]** | ***U* (*z*) / *t* (df)** | ***p*** |
| Age^1^ | 1.89 (1- 2.44; .31) | [1.78, 2.00] | 1.80 ( 1.23-2.43; .34) | [1.69, 1.91] | -1.16 (66) | .250 |
| Current CAARMS severity ^2,a^ | 6.03 (2.45-8.60; 1.67) | [5.42, 6.62] | 4.47 (0-9; 1.90) | [3.86, 5.08] | -1.55 (62) | **.001** |
| Current CAARMS Aggression severity ^a^ | 8.83 (0-16; 5.35) | [6.83, 10.83] | 7.32 (0-18; 5.33) | [5.46, 9.18] | 592. 50 (1.12) | .263 |
| Current CAARMS Suicidality severity ^a^ | 8.80 (0-24; 6.59) | [6.33, 11.26] | 5.65 (0-20; 6.11) | [3.52, 7.78] | 656.50 (2.01) | .044 |
| Lifetime CAARMS severity ^a,b^ | 47.67 (10-76; 20.10) | [40.16, 55.17] | 40.91 (1-92; 26.72) | [31.44, 50.38] | 584.50 (1.23) | .218 |
| Note. M = Mean; SD = standard deviation; *U* = Mann–Whitney test statistic; df = degrees of freedom; BCa95% of Mean Difference is based on bootstrapping with N = 2000 samples; LL= lower limit; UL= upper limit; CAARMS = Comprehensive Assessment of At-Risk Mental States – Short form.  ^1^Transformed using the reversed square-root of the values adding a constant of 1  ^2^Tranformed using the square-root of the values  ^a^*N* = 4 missing from the voice-hearing group  ^b^*N* = 1 missing from the comparison group.  For the parametric test estimates the means and independent t-test results are based on BCa95% bootstrapping with N = 2000 samples; For the non-parametric tests the CIs are based on 95%CI. Bonferroni-corrected critical p-value = .002. Value in bold font highlights the statistically significant effect. | | | | | | |

Table 3. Between-group differences in nominal demographic and clinical variables for the voice-hearing (N = 34) vs. comparison (N = 34) groups.

|  | **Voice-hearing (N = 34)** | **Comparison group**  **(N = 34)** |  |  |
| --- | --- | --- | --- | --- |
|  | **N ( Valid %)** | **N (Valid %)** | **Likelihood ratio χ^2^ (df)** | ***p*** |
| Female | 25 (73.5%) | 25 (73.5%) | 3.02 (2) | .459 |
| White British | 29 (85.3%) | 32 (94.1%) | 1.48 (1) | .427 |
| Limited day-to-day activities due to disability^a^ | 6 (17.6%) | 7 (21.2%) | .14 (1) | .765 |
| CAMHS | 28 (82.4%) | 27 (79.4%) | .09 (1) | 1 |
| Major Depressive Episode^b^ | 9 (28.1%) | 7 (20.6%) | .51 (1) | .57 |
| Panic Disorder ^c^ | 12 (38.7%) | 6 (17.6%) | 3.63 (1) | .095 |
| Agoraphobia *^c^* | 9 (29%) | 4 (11.8%) | 3.07 (1) | .121 |
| Social Anxiety*^c^* | 19 (61.3%) | 9 (26.5%) | 8.18 (1) | .006 |
| Obsessive compulsive disorder *^c^* | 6 (19.4%) | 6 (17.6%) | .03 (1) | 1 |
| Post-traumatic stress disorder ^a,^*^c^* | 7 (22.6%) | 3 (9.1%) | 2.25 (1) | .178 |
| Alcohol Use disorder 12 months^c^ | 7 (22.6%) | 5 (14.7%) | .67 (1) | .527 |
| Substance Use Disorder 12 months^c^ | 7 (22.6%) | 12 (35.3%) | 1.28 (1) | .289 |
| Anorexia Nervosa Restricting^c^ | 1 (3.2%) | 1 (2.9%) | - | - |
| Bulimia Nervosa^c^ | 4 (12.9%) | 0 | 6.21 (1) | .046 |
| Binge Eating ^c^ | 0 | 3 (8.8%) | 4.02 (1) | .24 |
| Generalised Anxiety Disorder^c^ | 2 (6.5%) | 7 (20.6%) | 2.88 (1) | .153 |
| Note*.* CAMHS = Child and Adolescent Mental Health Services; CAARMS = Comprehensive Assessment of At-Risk Mental States – Short form.  ^a^*N* = 1 missing from the comparison group  ^b^*N* = 2 missing from the voice-hearing group  ^c^*N* = 3 missing from the voice-hearing group.  Valid % represents percentage of participants with the available data; All tests were run with untransformed variables; For the likelihood ration chi square exact sig (2-sided is reported). All MINI Diagnosis except for the Alcohol and the Substance Use disorders refer to current research diagnoses. Bonferroni-corrected critical p-value = .002. | | | | |
